# Seasonal Changes in Hemolymph Protein Level and Hypopharyngeal Gland Size Depending on Age and In-Nest Location of Honeybee Workers

**DOI:** 10.3390/ani14030512

**Published:** 2024-02-04

**Authors:** Jan Musila, Antonín Přidal

**Affiliations:** Department of Zoology, Fishery, Hydrobiology and Apidology, Faculty of AgriSciences, Mendel University in Brno, Zemědělská 1, 613 00 Brno, Czech Republic

**Keywords:** Apis mellifera, physiology, age-polyethism, biomarkers, worker, distribution, sample

## Abstract

**Simple Summary:**

A honeybee colony is a complex aggregate of individuals that behaves as a unified organism regulated by division of labor among worker bees. It is closely related to the chronological and, above all, biological age of the workers. Their biological age can be estimated using biomarkers. The objective of this study is to monitor changes in these biomarkers (hypopharyngeal gland size, total hemolymph protein content) during the season in relation to worker age. Both biomarkers showed seasonal impacts in workers when their age was known. We aimed to clarify whether the worker nest distribution was age-dependent with a chronological/biological aspect; therefore, we analyzed workers in relation to their in-hive positions. The biologically younger ones of the same age were positioned predominantly in upper position and vice versa. Their total hemolymph protein content was significantly higher compared to those positioned below, and a strong total hemolymph protein content correlation was observed between them. Moreover, an in-hive, downward shifting mechanism was noticed as workers aged. Therefore, we propose a setup of starters and mating hives for young bees, preferably from the honey chamber. For the whole-colony condition estimation, according to physiological analyses of its workers, it is essential to sample workers whose age is known. Storage of diluted hemolymph samples before performing an assay is feasible if necessary, without the risk of changing the protein content within a month.

**Abstract:**

A honeybee colony, as a super-organism, is regulated through age-polyethism. A honeybee worker’s age is considered by means of a chronological and biological approach. The biological age is estimated with physiologically related biological markers, e.g., total hemolymph protein content (THP) and hypopharyngeal gland size (HGs), which also vary seasonally. Contemporary insights into the age-related spatial workers’ distribution within the hive nest space regarding biological age are insufficiently clarified. This study aimed to monitor changes in selected physiological markers during the entire season in relation to worker age and their spatial position in the hive nest. THP content and HG size analysis was performed in nine colonies for the entire season to compare the physiological markers within and among the groups of the workers whose ages were known and sampled in different hive parts. Seasonal impact on the biomarkers’ development was confirmed in known-age workers. In the case of HGs, this impact was the most apparent in 4- and 5-week-old workers. For THP, the seasonal impact was the most obvious in 2-week-old workers. The highest THP was found in 1- and 2-week-old workers during the entire season. Biologically younger workers of the same age were located predominantly in upper hive parts consistently throughout the year and vice versa. These workers showed significantly higher THP in comparison with those sampled below. Regarding the chronological age, the downwards, spatially shifting mechanism of workers within the hive nest while they aged was characterized. We recommend storage of diluted hemolymph samples up to one month before performing an assay if necessary. The physiological context, relation to division of labor and benefits for beekeeping practices are discussed.

## 1. Introduction

A honeybee colony is a complex system—a superorganism with cohort differentiation and a high degree of age-dependent division of labor [1,2,3]. In a colony, each task demands a specific anatomical and physiological state and behavioral competencies of the individual. The queen ensures the unity of the colony [4]. For honeybee workers, temporal/age-polyethism (division of labor) is characteristic. Workers perform various tasks as they age to meet the needs of the colony [5]. Not only behavioral differences but also physiological ones are recognized among bees of different ages [5,6,7,8,9]. Newly emerged bees tend to in-hive duties (hive bees), e.g., cleaning, brood and queen feeding, building, food receive and processing, etc. [10]. Nurses consume huge amounts of pollen as the main source of protein to hypertrophy their hypopharyngeal glands (HG) [11,12,13]. A fundamental behavioral change is observed at the age of approximately 3 weeks, when hive bees turn into foragers performing out-hive tasks, e.g., food collecting and colony guarding [2,14,15]. Foragers are characterized by an atrophied HG [11,16]. Generally, a worker’s physiological status within the age-polyethism corresponds to the values of physiological markers, e.g., the hypopharyngeal gland size, protein content in hemolymphs [8], antimicrobial activity of hemolymphs [17], juvenile hormone titer and vitellogenin in hemolymphs [8,18] and expression of the adipokinetic hormone receptor [19].

The workers’ role within the colony is closely related to their physiological status and depends on the colony’s demography [18,20], size of the colony [21] and its development [21,22] as well as on the season [23]. Flexibility is characteristic of the division of labor [13,14], so the workers of the same age could perform different roles and *vice versa*. Due to that fact, bee age is divided into chronological and biological age [19] to distinguish workers’ different physiological statuses. “The chronological age means the time elapsed since birth, hatching or another definitive early life event. Biological age refers to an animal physiological status relative to the normal developmental and post-developmental biological processes that occur throughout its lifespan. Biological age can be determined by key life-stage events … and measured by biomarkers.” [24]. Worker life-stage events are, for example, age at the onset of foraging, and biomarkers are, for example, vitellogenin and the adipokinetic hormone receptor [19]. The study by Alaux et al. [19] estimated the biological age of bees on the basis of the relationship between age and biomarkers to clarify whether the bees were exposed/not exposed to stressors. Thus, studies based on sampling workers whose age was not known [17,25] cannot be interpreted to either biological age or age-polyethism or colony demography. Proportions of biologically young and old workers in such bee samples were unknown and most likely differed between individual samples.

Hive bees show well-developed HG and high protein contents in comparison with foragers. That is associated with their high pollen digestion and secretion of protein-rich brood feed secrets, such as bee-milk and royal jelly [6,10,11,26,27,28,29,30]. Changes in nutrition and the division of labor also correlate with the substances circulating in the hemolymph and the size of HG [16,31]. Hive workers with the highest rates of protein synthesis also exhibit the highest protein levels in the hemolymph, but they may not always show the largest HG [28,32]. In foragers, hemolymph protein content reduces and HG shrinks and produce sugar-cleaving enzyme α-glucosidase [20,32,33,34,35,36]. The total hemolymph protein content is low in newly emerged workers and rises with their age and pollen consumption [37,38,39]. Workers start to eat pollen soon after emergence, reaching a peak at about 5 days of age [40]. Pollen consumption is then diminished at 8–10 days [41]. Similarly, newly emerged workers’ HG is light and soon become heavy in 3-to-21-day old hive bees [8].

The size of the HG is one of the apparent physiological markers in worker honeybees. It is an exocrine gland physiologically dependant on the division of labor [42]. In foragers, the HG is controlled by the juvenile hormone [4,8,43] and brood pheromone [1,9,44].

Analysis of the THP also ranks among the standard methods of determining a worker bee’s physiological state [45]. One of the main hemolymph proteins is vitellogenin, as it is an important storage glyco-lipo-protein highly concentrated in long-lived bees [8,46], which is implicated in the regulation of immune integrity [47] and providing protection from oxidative stress [48]. The hemolymph vitellogenin titer distinctly increases in newly emerged workers and reaches its peak in about 12-day-old summer bees and then decreases [7,49]. The winter long-lived workers show a high vitellogenin titer up to twice as high as that in 12-day-old summer workers [46].

Plasticity in age-related division of labor is at least partially controlled by social factors. Behavioral development can be accelerated, delayed and reversed by manipulating colony age structure. Confining foragers to colonies results in delayed behavioral development. Conversely, depleting foragers results in accelerated behavioral development [18]. Similarly, changes in age-related division of labor corresponds with changes in the spatial distribution of the workers performing certain tasks within the hive nest [50]. During the course of the experiments for the study by Přidal and Šustek [51], it was found that the spatial distribution of the workers within the hive nest was not random but correlated with their age (unpublished results).

The season-based variations in worker honeybee physiology have been studied before [17,25], albeit without taking the chronological or biological age of workers into consideration. Therefore, we intended to monitor changes in selected physiological markers during the season precisely with regard to worker age. In addition, to evaluate the hypothesis that the distribution of workers throughout the nest is age-dependant, chronologically and/or biologically, we analyzed workers with respect to their spatial position in the hive nest. Moreover, we verified protein stability in hemolymph samples, which means the storability of the hemolymph samples before analysis with regard to the applied methodology.

## 2. Materials and Methods

### 2.1. Selection and Treatment of Experimental Bee Colonies

The experiment was conducted under conditions of a temperate climate in Brno, Czech Republic (2021), using honeybee (*A. mellifera* L.) colonies of European origin and the strain VIGOR^®^, and the open-air apiary consisted of a total of 42 bee colonies kept in box hives. Almost every hive was assembled from two brood chambers with frames set to the cold way (10 frames 370 × 300 mm in each), one super placed under the lower edge of the brood chamber and 1–2 honey supers placed above the top brood chamber (each super consisted of 10 frames 370 × 170 mm). Twenty-one bee colonies were selected and sorted into three strength categories (7 colonies in each) according to the subjective mode of the colony strength assessment to preserve the social cohesion according to BEEBOOK [52] in mid-April 2021. Strength category 1: a weak colony occupying 3 to 4 combs, consisting of approximately 10,000 bees; strength category 2: a medium colony occupying 5 to 6 combs, consisting of approximately 15,000 bees; and strength category 3: a strong colony occupying 7 to 8 combs, consisting of approximately 20,000 bees. These strength categories were chosen to minimize the possible effect of colony strength on the character of the individual worker’s biomarkers. This aspect has not been generally applied in the studies [8,17,25]. The queens heading experimental colonies were genetically closely related (inbred population). All colonies had brood in all stages of development and performed standard brood activity without signs of discontinuous sealed brood and preparation for swarming. Only colonies in a similar state of health—without obvious clinical disease symptoms and with a low ectoparasitic mite (*Varroa destructor*) infestation rate—were used. *Varroa destructor* infestation rates were controlled using oxalic acid (OA) treatment (OA glycerine solution in cellulose long-term carriers in summer, OA sugar syrup solution trickling in winter). Therapeutic mite fall was monitored at the diagnostic hive bottom in all hives, since the experimental colonies were selected in spring, until the end of all experiments.

### 2.2. Worker Bees’ Biological Age Marker Development during the Season—Experiment 1

The goal of this experiment was to monitor changes in the total hemolymph protein (THP) content and hypopharyngeal gland (HG) development within the entire season of 2021 from the end of April to the end of July in worker bees that emerged on 24 April, 29 May and 24 July to make cohorts of workers from different parts of the season (i.e., three seasonal cohorts: spring, early summer and late summer). Each cohort was represented again by three colonies of all strength categories: weak, medium and strong. Spacing among hives was sufficiently large to prevent drifting bees. In order to obtain 200–300 worker bees of the same age, 1–3 brood combs containing predominantly late-stage pupae shortly before emerging were taken from each colony without adults. The brood combs were placed into an incubator at 34.5 °C and 60% humidity in a pouch from meshes to preclude the mixing of bees from different colonies. After 20 to 24 h, 200–300 emerged workers were marked on the mesothorax using water-based markers (POSCA pen, Japan) and returned to the maternal colony. Each cohort was paint-marked with a different color to distinguish the age/cohort of subsequently sampled bees from a colony.

For the total hemolymph protein assay and the hypopharyngeal gland development evaluation, workers were sampled according to the schema shown in Table 1.

Larger samples of the late summer cohort were collected because the weak colony did not live to this part of season. Smaller samples were made from the weak colony of the spring cohort due to a higher rate of worker disappearance/death, which was typical, especially for weaker colonies in the springtime. A similar reason for the smaller number of samples occurred in the 4th and 5th weeks when it was not possible to locate the complete number of workers due to their older age. For example, the spring workers did not reach the 5th week of age in any colony strength. Workers were sampled into plastic vials that were placed into a refrigerator (4 °C) to immobilize the bees for subsequent hemolymph collection and decapitation, as described below.

### 2.3. Worker Bee Position within the Hive Nest—Experiment 2

The goal of this experiment was to demonstrate the physiological statuses (biological age) of worker bees in relation to their position within the hive nest (in-hive position). The experiment was carried out in two different seasonal intervals: (a) the initial period of the season from April to August 2021, i.e., the growth and productive part of the season; (b) the final period of the season from July to November 2021, i.e., processing supplemented sugars to the winter stores and preparing for the wintertime. The seasonal division was made to facilitate experimental operations. Season-long recording of color-marked workers in the same colony from April to November was impossible since the total number of age cohorts to be monitored was too high (up to 17 cohorts) compared to the available range of basic colors that are easily distinguishable from one another. Three-week-old workers were sampled during the initial period because workers have faster aging dynamics and hardly ever live longer than five weeks, unlike workers that emerged in the final period [18]. One- and two-week-old workers are always house bees of low biological age with fully developed HG. Thus 5-week-old bees were sampled in the final period, when 3-week-old workers tended to be biologically younger (with delayed behavioral development), and their ethological differentiation (transition to foragers) came at a higher chronological age [18,51].

The experiment in both periods was carried out in nine colonies every time (three colonies in each of the three strength categories as described above), i.e., in eighteen colonies in total. The brood combs were placed in the incubator every 12th day throughout the experiments. In this way, nine seasonal cohorts of workers were chronologically created in the initial period and eight seasonal cohorts were created in the final period. Each seasonal cohort consisted of 200 color-marked individuals, which, after marking, were immediately introduced back to the maternal colony. The bees were sampled for the evaluation of the total hemolymph protein. First of all, the hives were disassembled before sampling the bees, i.e., supers were put aside separately to prevent the random movement of bees among supers as a result of a colony disturbance. Each bee sample contained five worker bees of a given seasonal cohort (color). The samples were collected throughout the course of the experiments (sampling every 3rd week of age in the initial period and every 5th week of age in the final period). We aimed to sample at two hive positions: (a) upper position = top parts of the honey chamber—the uppermost super; (b) lower position = under the bottom border of the brood chamber—the lowest super. The bees in the winter cluster had to be sampled without position distinction. The bees were put into a refrigerator (4–8 °C) immediately after sampling to be immobilized for subsequent assays.

To record the positions of bees of known age within a hive nest, three medium and three strong colonies were monitored in 12-day intervals from 15 August to 2 October 2021 (i.e., the final period of Experiment 2 in the 3rd emerged seasonal worker cohort). The weak colonies were excluded due to presumed insufficient hive nest volume for the accurate evaluation of the workers’ in-hive positions (further details in the discussion). The inspections were performed at the ages of 1, 13, 25, 37 and 49 days. The supers of a hive were disassembled from each other before the colony inspection to prevent a free accidental migration of disturbed bees among supers. Only afterwards was the colony inspected and the position of individual marked bees within the hive nest (under the brood chamber, the brood chamber itself and the honey chamber) recorded precisely. The inspected combs were put into another empty super to avoid mixing recorded bees with unrecorded ones in the same super.

**Figure 1:**
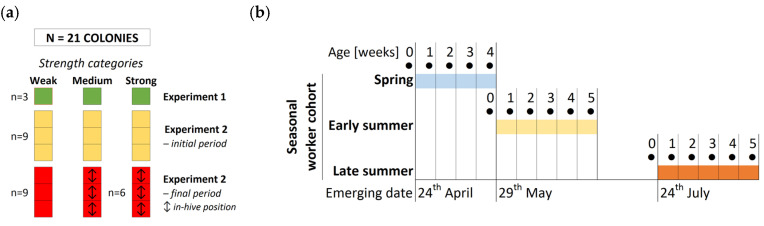

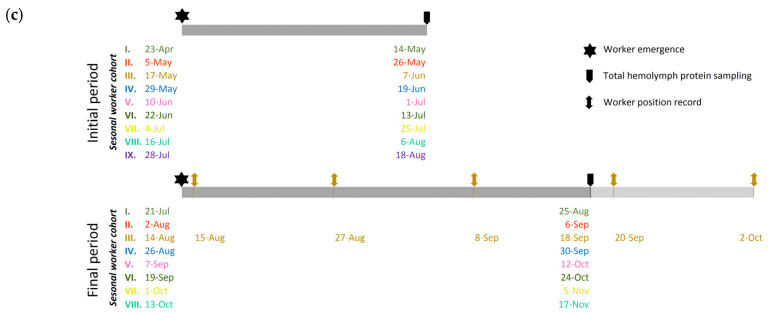
Schematic diagram of the experiments: (**a**) Number and composition of colonies in all experiments; (**b**) Experiment 1—seasonal monitoring of THP and HGs depicted according to seasonal workers’ cohorts and sampling ages (●); (**c**) Experiment 2—the initial and the final periods and dates of the emerging and sampling of workers in individual cohorts and during the season; colors correspond with the marking of bees in the individual workers’ cohorts.

The setup of all experiments is depicted in the schematic diagram below (Figure 1).

### 2.4. Hypopharyngeal Gland Dissection

Each worker bee was decapitated before hemolymph collection, and its head was put into a 0.5 mL Eppendorf tube containing 30 µL of 75% ethyl-alcohol to be preserved at room temperature until dissection. HG was dissected by cutting off the forehead cuticle with a micro-scalpel and ablated together with tracheal sacs with tweezers. For each worker, the diameters of approximately 10 neighboring pseudoacini of right HG were measured in µm perpendicular to the longer axis of the oval acinus [16,42,53]. This was conducted with an ocular micrometer using Carl Zeiss Jena binocular microscope (magnification 90’).

### 2.5. Hemolymph Collection

Hemolymph was collected individually from a drop squeezed out of the thorax at a volume of 1 µL using a micro-capillary pipette after the worker’s abdomen had been ablated with scissors. The hemolymph of each worker was immediately pipetted separately into a 1.5 mL Eppendorf tube containing 49 µL of phosphate buffer (pH = 7) and immediately vortexed to improve the stability of the hemolymph proteins and frozen (−20 °C), and it was analyzed no later than 1 month after collection. The phosphate buffer (pH = 7) was prepared by mixing di-Sodium hydrogen phosphate anhydrous (11.876 g/L) and Sodium phosphate monobasic monohydrate (9.078 g/L).

### 2.6. Total Hemolymph Protein Quantification

The Bradford assay was applied [54]. In total, 5 µL of 50× diluted hemolymph sample was mixed with 250 µL of Bradford reagent (Sigma-Aldrich, Merck, St. Louis, MO, USA). The absorbance at 595 nm was measured using a FUOstar Omega plate reader (BMG Labtech, Ortenberg, Germany) after 5 min of incubation at room temperature. The total protein concentrations were calculated using a calibration curve. The calibration curve was made separately for each micro-titration plate, using bovine serum albumin (Sigma Aldrich) as standard. Each sample was measured three times.

### 2.7. Hemolymph Storage

The stability of the protein in the collected bee hemolymph has not been studied yet. Therefore, we tested changes in protein content as a function of time during storage at different temperatures with and without N-phenylthiourea to ascertain whether the protein hemolymph assay could be postponed until at least one month after hemolymph collection and whether N-phenylthiourea is essential for the assay accuracy.

Approximately 260 worker bees of unknown age were shaken off a comb into a plastic vial from a single colony in Brno on 1st July 2020 for both the storage methods. Immediately after shaking, they were placed into a refrigerator (4 °C) to be immobilized before hemolymph collection, as described above.

N-phenylthiourea (PTU) is recommended to prevent hemolymph melanization [55]. PTU solution was prepared by mixing PTU powder (purity ≥ 98%) and MilliQ water (demineralized water, APS Water Services Corporation, Los Angeles, CA, USA) at a concentration of 1 µg PTU per 1 µL of MilliQ. All chemicals were purchased from Sigma-Aldrich (St. Louis, MO, USA). The PTU solution was subsequently homogenized via ultrasound and vortexed until the PTU crystals totally dissolved.

A source sample for this storage experiment was prepared as a pooled sample from 120 µL of hemolymph and 30 µL of PTU solution pipetted into a 1.5 mL Eppendorf tube, vortexed and used to prepare a total of 96 subsamples for chronological (at 0 and 24 h, 7, 14 and 28 days after hemolymph collection) and temperature (−80 °C, −20 °C and 4 °C) assays. Each subsample was created by mixing 1.25 µL of the pooled sample with 49 µL of phosphate buffer and vortexing. Six subsamples were prepared for each temperature and time (six repetition in each variant).

We did not observe any melanization in a preliminary assay. We supposed that it was caused by a high dilution of hemolymph (dilution factor 50). This experiment verified that PTU can be excluded from the above-described assay without affecting the accuracy.

A source sample for this storage experiment was prepared as a pooled sample from 120 µL of hemolymph pipetted into a 1.5 mL Eppendorf tube, and it was used for preparing, again, a total of 96 subsamples stored at the same temperature and time intervals. Each subsample was created by mixing 1 µL of the pooled sample with 49 µL of phosphate buffer.

Firstly, bees were taken out of the vial one by one, and 1 µL of hemolymph was taken from each of them according to the methodology described in Section 2.5. and pipetted into a 1.5 mL Eppendorf tube until 120 µL of hemolymph was collected (pool sample) and then homogenized using a Vortex-Mixer (PV-1 Grant-bio, Fischer Scientific, Loughborough, UK).

Secondly, 1 µL of hemolymph from the pooled sample was pipetted to a 1.5 mL Eppendorf tube containing 49 µL of phosphate buffer (dilution 50×) and vortexed.

### 2.8. Statistical Analysis

Statistical analysis was performed with the null hypothesis that the differences in the content of THP or HG size between/among workers were statistically significant when *p* < 0.05. Data were analyzed using parametric tests: one- (differences in THP content among seasonal cohorts) or two-way ANOVA (hemolymph storability at different temperatures) and the Student *t*-test, with the verification of the homogeneity of variances (F-test), independent two-sampled and two-tailed, were performed to analyze the effect of the workers’ positions in the colony nest.

## 3. Results

### 3.1. Monitoring the Sesonal Biomarkers—Experiment 1

**Figure 2:**
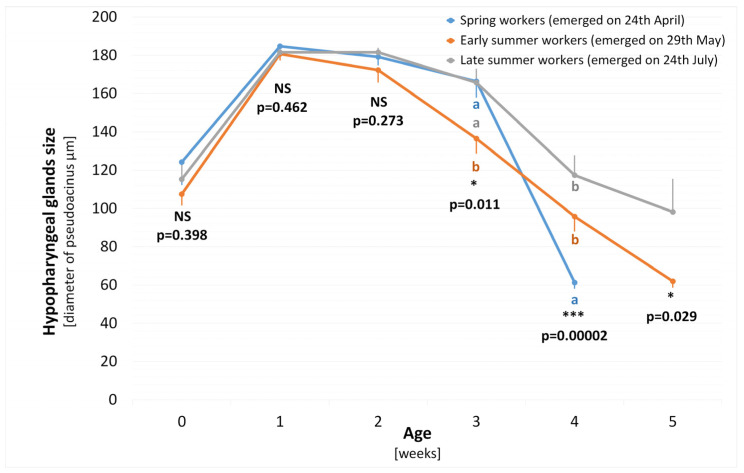
Hypopharyngeal glands size (mean diameter of pseudoacini) among the seasonal worker bee cohorts (spring, early summer and late summer workers) in relation to their age (0–5 weeks). Arithmetic mean ± SEM (bars), three-sample comparisons tested with one-way ANOVA, and different letters show significant differences among seasonal cohorts (post hoc Tukey test *p* < 0.05); two-sample comparison tested with a *t*-test. * *p* < 0.05; *** *p* < 0.001; NS non-significant.

**Figure 3:**
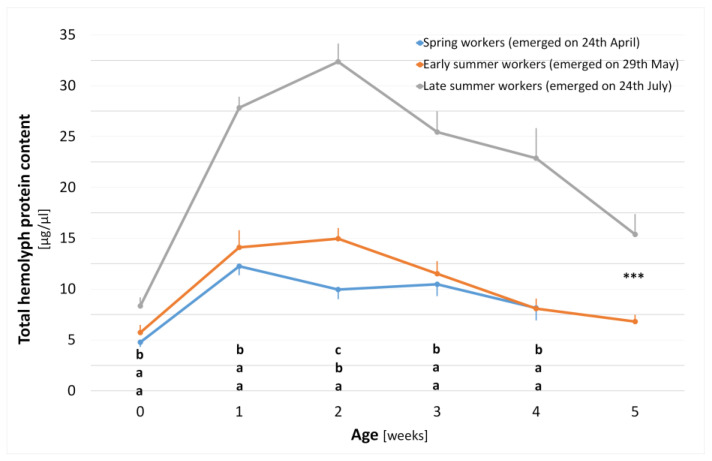
Ontogeny of THP content within a season in three seasonal worker cohorts (spring, early and late summer). Arithmetic mean ± SEM (bars), three-sample comparisons tested with one-way ANOVA (F_2,41_ = 8.18, *p* = 0.001; F_2,75_ = 51.32, *p* < 0.001; F_2,80_ = 81.01, *p* < 0.001; F_2,80_= 31.10, *p* < 0.001; F_2,40_ = 16.84, *p* < 0.001 respectively), and different letters show significant differences among seasonal cohorts (post hoc Tukey test *p* < 0.05); two-sample comparison tested with a *t*-test (t_28_ = 4.688, *** *p* < 0.001).

The seasonal development of HG size and THP content in spring (emerged on 24 April), early summer (emerged on 29 May) and late summer (emerged on 24 July) worker bee cohorts are displayed in Figure 2 and Figure 3, respectively.

#### 3.1.1. Hypopharyngeal Gland Size Development

Newly emerged workers showed lowered HG development in all the seasonal cohorts, and differences among them were not statistically significant (ANOVA F_2,41_ = 0.9418, *p* = 0.3981). Uniformly, fully developed HG in all the seasonal cohorts was found in 1- and 2-week-old workers. The first decline in HG development was found in 3-week old workers in the early summer cohort, with confirmed statistical significance (ANOVA F_2,80_ = 4.7756, *p* = 0.0110). In the following weeks, i.e., in the workers’ 4th and 5th weeks of age, only decreasing HG development was recorded in all the seasonal cohorts. At four weeks old, the differences among the seasonal cohorts are significant (ANOVA F_2,44_ = 13.5700, *p* = 0.00002), and the Tukey post hoc test proves significance between spring and early summer workers and spring and late summer workers. The differences between early and late summer workers are not significant (post hoc *p* = 0.1066). The spring workers did not reach the 5th week of age in any colony strength. The differences in HG development between the early and late 5-week-old summer workers are also significant (*t*-test, *t*-value_25_ = 2.324, *p* = 0.0293).

#### 3.1.2. Total Hemolymph Protein Content Development within a Season

Newly emerged workers showed the lowest values of THP content and the smallest differences among all the seasonal cohorts (ANOVA F_2,41_ = 8.1835, *p* = 0.001). The THP content sharply increased in all the seasonal cohorts and reached its maximum in one-week-old spring workers and two-week-old early and late summer workers. The later the workers emerged during the season, the higher maximum value of THP content they reached within their ontogeny (12.25, 14.96, 32.36 µg/µL; spring, early summer and late summer worker cohorts, respectively). The highest differences were found at the age of two weeks, where all seasonal cohorts significantly differed from each other (ANOVA F_2,80_ = 81.0067, *p* < 0.001, tested using the post hoc Tukey test). In the following weeks, only decreasing contents of THPs were found until the marked workers fully disappeared from the colonies. THP content in the late summer worker cohorts significantly differed from both remaining cohorts throughout the entire experiment. The difference in THP content between the early and the late summer workers at 5 weeks of age was significant (*t*-test, *t*-value_28_ = 4.688, *p* < 0.001). The spring workers did not reach 5 weeks of age in any colony strength.

### 3.2. Worker Bee Position within the Hive Nest—Experiment 2

**Figure 4:**
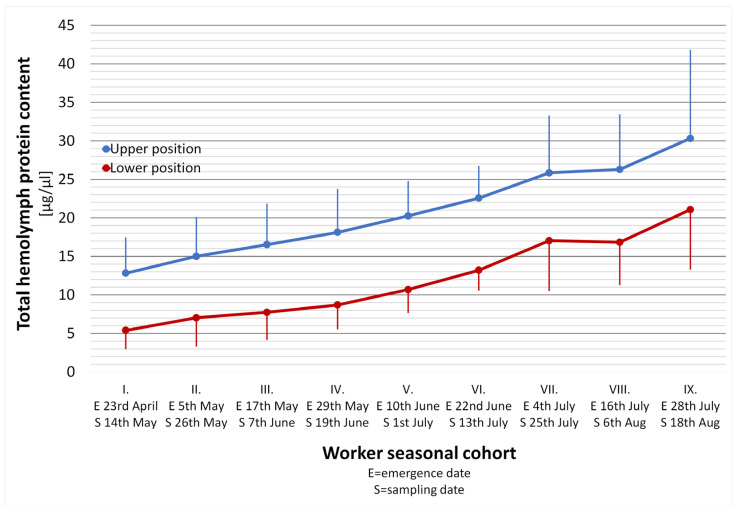
The total hemolymph protein content in three-week-old worker bees of nine worker seasonal cohorts that emerged in twelve-day intervals from 23 April to 28 July (the initial period of the season) in relation to worker position in the hive nest. The dots represent arithmetic means (n = 45; five workers from each of the nine colonies) ± SD (bars). Differences between the upper and lower position in each age cohort are statistically significant in all cohorts (*t*-test, *p* < 0.0001).

**Figure 5:**
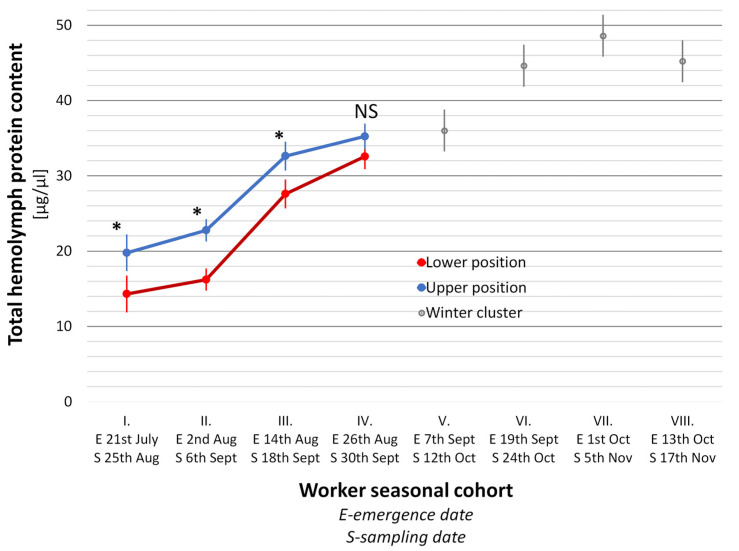
The total hemolymph protein contents in five-week-old worker bees of eight worker seasonal cohorts that emerged in twelve-day intervals from 21 July to 13 October (the final period of the season) in relation to worker position in the hive nest. The colored dots represent arithmetic means (n = 45) ± SD (bars) before the formation of the winter cluster. Differences between upper and lower positions are statistically significant in the first three cohorts (*t*-test, * *p* < 0.05), and, in the 4th cohort, non-significant (NS *p* = 0.270). Seasonal cohorts 5–8 were not distributed throughout the entire hive nest but only in the winter cluster represented by grey dots (n: 50, 90, 90 and 90, respectively).

**Figure 6:**
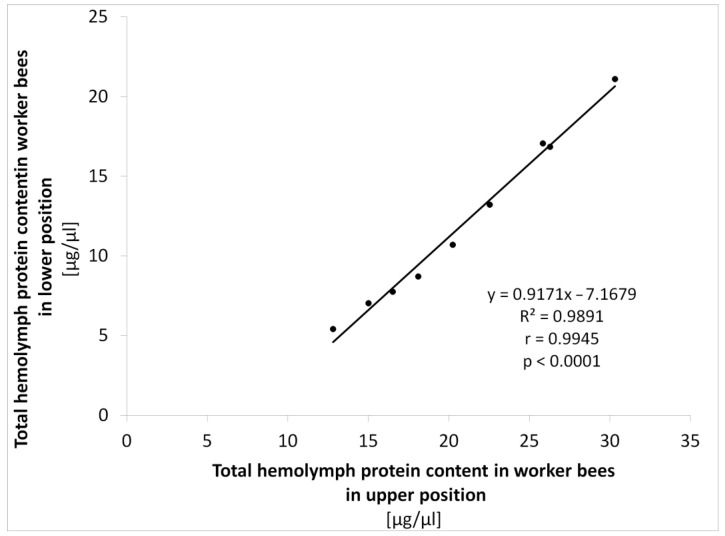
Regression analysis of the total hemolymph protein content in the upper and lower located workers within the initial period of the season and regression equation, and the coefficients of determination (R^2^) and correlation (r) and *p*-value are given.

The THP development trends in relation to worker in-hive position are shown in Figure 4 (the initial period of the season) and Figure 5 (the final period of the season). The level of THP content in three-week-old workers (the initial period of the season) was strictly dependent on their position within the hive nest in all the seasonal cohorts (*t*-test, *p* < 0.0001). The THP content showed a similar growth trajectory in both upper and lower workers’ positions, except for the period between the 7th and 8th seasonal cohorts. The only scanty decrease was recorded for the lower located workers in this period, while the THP content in upper located workers continued rising slightly. The mean difference of THP content between the upper and the lower located workers was 8.9 µg/µL ± 0.74 SD for the entire initial period (Figure 4). The THP contents between the upper and the lower located workers are highly interdependent, as expressed with the regression analysis (Figure 6). Gradually and slowly increasing levels of THP content in the upper located workers were followed by a very similar trend in the lower located workers, as is expressed by the high coefficients of determination and correlation.

The THP content increase also continued in both the upper and lower located workers in the final period of the season. Even at this stage of the experiment, the level of THP was still strictly and significantly dependent on worker position (*t*-test, *p* < 0.05) until the winter cluster was formed (Figure 5). The only difference between the upper and lower positions in the 9th seasonal cohort was not significant (*t*-test, *p* = 0.270). The correlation between the upper and the lower located bees was again positive and very high in all tested seasonal workers’ cohorts (1st–4th, r = 0.9933, *p* < 0.01). Starting with the 5th seasonal cohort, the workers’ position could not be distinguished because of the winter cluster formation (grey dots in Figure 5).

**Figure 7:**
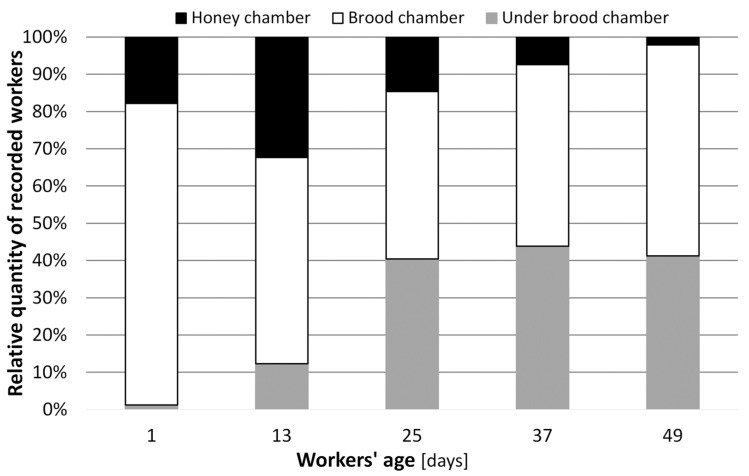
Average worker positions within the hive nest of six colonies (n = 6) during the aging of the identical worker cohort that emerged on 14 August 2021.

The positions of workers from the same worker-age cohort that emerged on 14 August 2021 within the hive nest as they aged are depicted in Figure 7. The majority of the newly emerged workers assembled on brood combs in the brood chamber (81%), and some of them assembled on honeycombs (18%); only 1% was located under the brood chamber. Twelve days later, 33% of the age cohort members were found in the honey chamber, with 55% in the brood chamber and 12% under the brood chamber. At an age of 49 days, most of this age cohort’s members (57%) were recorded in the brood chamber. In general, the older the workers were, the more they were recorded under the brood chamber and the less frequently they were recorded in the upper parts of the hive nest. In other words, as the workers aged, they disappeared from the top hive nest parts and shifted to the lower positions of the hive nest (the gray parts of the columns in Figure 7 enlarge with increasing age of the workers), and to find a forager in the honey chamber was quite exceptional.

### 3.3. Hemolymph Storability

**Figure 8:**
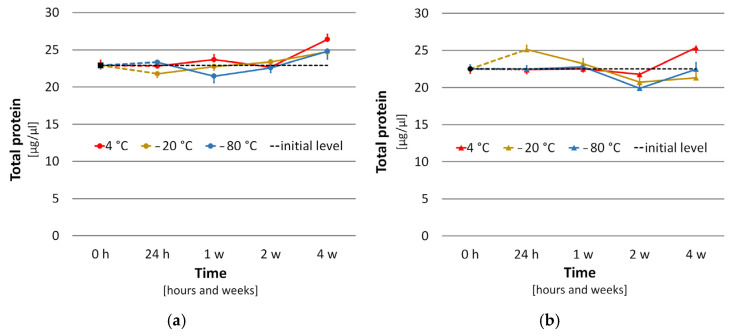
THP content in (**a**) PBS (●) and (**b**) PBS + PTU (▲) samples as a function of temperature and time (h = hours, w = week(s)); the dots represent means ± SEM (bars), n = 6; the black dot and dashed line show the initial level of THP.

The results showed statistically significant changes in THP contents over time in hemolymph (H) samples diluted in phosphate buffer saline (PBS) (F_2,4,75_ = 10.6963, *p* < 0.0001), as well as in PBS with N-phenylthiourea (PBS+PTU) (F_2,4,75_ = 9.5516, *p* < 0.0001). Conversely, the impact of the storage temperature on the stability of the THP content was not proved in either H+PBS (F_2,4,75_ = 1.8210, *p* = 0.1690) or H+PBS+PTU (F_2,7,75_ = 2.4372, *p* = 0.0943). The THP content varied negligibly (not more than 1.1 µg/µL among means) under different storage temperatures in both H + PBS samples (4 °C $\bar{x}$ = 23.9, c_v_ = 9%; 20 °C $\bar{x}$ = 23.2, c_v_ = 7%; −80 °C $\bar{x}$ = 23.1, c_v_ = 9%) and H+PBS+PTU samples (4 °C $\bar{x}$ =23.0, c_v_ = 8%; −20 °C $\bar{x}$ = 22.6, c_v_ = 11%; −80 °C $\bar{x}$ = 21.9, c_v_ = 8%) in comparison with the means for all measured values of each temperature data set (n = 24). The course of changes in THP content of H+PBS and H+PBS+PTU samples is displayed in Figure 8.

A more detailed analysis of the differences in THP content between fresh (control) and stored H samples in relation to time is presented in Table 2. The differences were not higher than 2.5 µg/µL. Only three statistically significant differences were confirmed: one in the 4th week of H+PBS storage and then on day one and in the 1st week of H+PBS+PTU storage.

Finally, the influence of PTU additive in PBS on differences in the THP contents was analyzed. The differences were calculated between the mean values of THP content in the stored samples (n = 12), on the one hand, and the fresh sample, on the other hand. The mean differences were PBS 0.6 µg/µL and PBS+PTU 1.2 µg/µL, and they were not significantly different (*t*-test; *p* = 0.303; *t*-Value_22_ = 1.055).

## 4. Discussion

Seasonality of biomarkers in honeybee workers has been studied in bees of unknown age [17,25]. However, biomarkers reflect not only the season but also the age-polyethism [19]. Analysis of biomarkers in unknown-age workers can only result in broadly averaged values with low applicability for any physiological indications/prediction. Thus, the objective of this season-long study was to monitor biomarkers in workers whose age was known. The results confirm that biomarkers (HGs and THP content) increase during the season, and workers reflect their biological age through their in-hive position within a fully structured hive.

### 4.1. Biological Age and Worker Bee Position in the Colony

Alaux et al. [19] created a biological age prediction model from the relationship between a workers’ age and biomarkers of their task specialization. Analogously, we sampled workers of known age and focused on the following biomarkers: (i) THP content and HGs —to estimate the biological age development of workers’ cohorts representing all-season-long standard biomarker values; (ii) THP contents and their positions within the hive nest—to determine differences in biological age among workers of the same chronological age in the hive nest throughout the season. We found significant differences in the THP content according to their position within the hive nest. Workers sampled under the brood chamber had a significantly lower THP content on average. Biologically younger bees were found to be located in the upper parts of the hive nest, unlike the biologically older bees that mostly appeared in the lower parts of the hive nest. In accordance with Free [50], the present study proves that newly emerged bees appear on brood combs, and biologically young, 13-day-old nurses were predominantly recorded in the brood and the honey chamber. Contrary to our findings, Free [50] monitored larger numbers of bees older than 15 days in stores in the upper parts of the hive nest. We recorded 25- to 49-day-old foragers gradually accumulating in lower parts of the hive nest on empty combs under the brood chamber, and many of them had pollen loads at the time of sampling. Therefore, we suggest that workers’ spatial distribution within the hive nest is based on behavioral polyethism (division of labor).

We suppose that this discrepancy in workers’ distribution according to their chronological age could have been caused by the controlled, non-natural structure of the small hive nests in Free’s experiment [50]. He applied only two supers, with honey stores being located both in the lower and upper ones, and the brood reached the lower edge of the hive nest; thus, a bottom zone with empty comb cells was missing.

Similarly, van der Steen et al. [56] used an extremely constricted hive space (a one-storey hive) where workers were evenly distributed on each frame no matter their age. Unlike both of these studies, we used fully spacious hives, enabling natural worker distribution on honey stores predominantly located in the top parts of the hive nest (honey chamber) and on the empty cells under the brood chamber. Thus, workers could spread in a structured manner within the hive nest according to their biological age and colony requirements throughout the year.

Young bees are needed to establish starters, package bees or mating hives/nuclei if good zootechnical practice is to be followed [57]. Thus, knowledge of the age–spatial distribution of workers within the hive nest is essential. According to a previous study [50], workers in the honey chamber are old (foragers); therefore, young bees (nurses) should be taken only from the brood chamber. This assumption is logical and, therefore, also generally recommended in beekeeping instructions [58,59,60]. However, we repeatedly found both chronologically and biologically older bees predominantly only in lower parts of the hive (in and under the brood chamber). In other words, the older the workers were, the lower down in the hive nest they were found, and vice versa; the biologically younger, but of the same chronological age (peers), workers were predominantly recorded in the upper parts of the hive nest. This fact can be considered in beekeepers’ practices. Taking workers from the hive is usually easier and safer when they are shaken from the honey chamber, especially if the queen is separated in the brood chamber under a queen excluder.

### 4.2. Hypopharyngeal Gland Size

However, with regard to biological age expressed by the size of the HG, Free [50] recorded more bees with fully developed HG on the stores and brood combs at all developmental stages than those with underdeveloped HG. Accordingly, we also recorded the majority of workers with developed HG in the brood chamber and on honey stores, whereas workers with underdeveloped HG were predominantly found under the brood chamber and absent in the honey chamber. Thus, Free’s [50] findings are inconsistent because they differ in terms of the chronological and biological ages. In agreement with Soudek [11] and later Fluri et al. [8], we observed, in 1- to 3-week old workers, fully developed HG, and newly emerged workers had underdeveloped HG. Pollen consumption is essential for HG development and increases until emerged workers become nurses [10,29,61]. The shrinkage of HG tends to start in 3-week-old workers [8,62], which was also confirmed by this study. The dynamics of HG development is changed in colonies with swarming fever, where fully developed HG can also be recorded in chronologically old bees in the 4th, the 5th and the 6th weeks of age [52,62]. A similar physiological phenomenon has been found in the autumnal transition of a colony to the production of the winter generation of workers [8]. In the present study, we found spring worker biological aging dynamics expressed through HG shrinkage to be the fastest of all the seasonal worker cohorts. Characteristic high-protein-secretory activity in the HG of nurses later dynamically changes to invertase activity during their transformation process into foragers accompanied by HG shrinkage in summer [33]. In the present study, the most underdeveloped HG were always monitored in workers with pollen loads. The spring worker cohort’s fast biological aging being closely linked to its short longevity might be caused by intensive brood rearing and rapid colony development after wintertime. This could explain why spring workers could not reach the age of 5 weeks in our experiment. Conversely, later in the season, workers’ lifespans extended to 5 and more weeks, and their HG shrinking dynamics slowed down. The longest lifespan was recorded in the late summer worker cohort, which might be linked to some pre-initiation of the formation of winter bee generation.

### 4.3. Total Hemolymph Protein Content

In accordance with previous studies [32,37,38,39], the newly emerged workers had the lowest THP contents of all measured bees in the experiment. This fact might be connected to the quality and quantity of their pre-emergence nutrition, supported by the findings from Kunert and Crailsheim [63], that the protein body content in newly emerged workers is dependent on the availability of food outside the hive. Workers raised under good pollen and nectar flow had more body protein than those raised under worse nutritional conditions. In the presented study, the peak of THP content was recorded in the 2-week-old workers, with the exception of the spring cohort, where the peak was achieved a week earlier, which is consistent with results from Basualdo et al. [39] that observed this in well-fed, caged workers. The dynamics of post-emergence THP content in adult workers depends on the character of post-emergence nutrition [39]. This may explain the THP content increase in workers over the course of 2 weeks after emergence in all observed seasonal cohorts. We found that these peaks were increasing within the season, and, similarly, THP content also grew in 3- or 5-week-old workers. This result, however, does not correspond with findings from Kodrík et al. [25], who monitored throughout the year. We assume this was caused by analysing workers of unknown age and, therefore, not considering age-polyethism in their experiment, as was also the case in the study from Kunc et al. [17]. However, it is necessary to know the exact age of the analyzed bees for physiological experiments [31] because physiological markers are not only nutritionally dependant but also age-dependant in the biological and chronological sense [19]. In both the studies [17,25], workers of an unknown age were collected either “from the same place on a specific frame in hive, where worker bee foragers should be predominantly” or “from the comb which was not part of the brood chamber but was located right next to it to avoid the sampling of newly hatched adult”’. Contrary to their prediction, the present study’s results correspond to the previous findings that “it is not possible to accurately collect individuals of known ages based on location within the colony” [64]. In a hive with constricted space (confined space without natural structure including the brood and honey chamber), workers of all ages were distributed evenly on each comb [56].

Regarding workers’ biological age, the previous study [19] created a very useful worker-biological-age prediction model to evaluate the condition of a colony infested with *Varroa destructor*. To estimate the biological age, a calibrated sample set of known-age bees was determined using biological markers (e.g., vitellogenin). This sample was represented by bees from only one emergence date but for the evaluation of all workers that emerged in different parts of the season. According to the present study, the results show significant differences in the dynamics of THP content across the season and among the seasonal workers’ cohorts. This is why we assume that the seasonality could play a role in such biological age prediction models.

As mentioned above, the average THP content in 3- or 5-week-old workers continued increasing throughout the season until pre-wintertime. Winter bees have high THP contents, which remain at a high level until the end of winter [8,65,66,67,68], and Wahl [69] also recorded larger protein contents in different body parts of young workers at the beginning of autumn compared to the summer generation. Foragers have the lowest THP contents of all workers [8]. This could explain the fast THP content decline in workers who emerged in the spring and early summer. Forager senescence is fast due to the intensive brood care duty and associated food collection duty performed in summer [70,71]. We assume that this late summer worker cohort was still under the influence of factors preventing the emergence of winter bees: (a) processing supplemented sugars provisioning winter stores [72,73], (b) pollen collecting [74] and, above all, (c) the brood pheromone [71]. The amount of reared brood is inversely correlated with worker longevity [23,70,75,76]. Once workers begin nursing, they have a high demand for pollen-derived nutrients to support brood-rearing physiology. Consequently, the amount of pollen available to workers has an enormous influence on the length of time that they can continue to nurse [77]. The condition of spring workers reared in colonies supplemented with pollen was studied by Mattila and Otis [77,78], who found that the supplemented colonies reared more workers, and these were longer-lived in the first year and shorter-lived in the second year of their experiments. Other parameters for worker bees reared in the colonies supplemented with pollen were similar between the years: the proportion of workers that survived was higher, the proportion of workers that foraged over time was lower, and these workers spent more time tending to the brood and had higher protein contents in their heads. As a consequence, more honeybee brood and subsequently emerging bees led to the accelerated behavioral maturation of bees. The initial larvae instars produce the pheromone E-β-ocimene [79]. Freshly emerged young bees produce a young bee pheromone, and its accelerating effect was demonstrated with methods of extraction [80] and, later, with gene expressions [81]. Moreover, they found that the brood and the young bees caused significant decreasing in gene expression levels for vitellogenin [81]. It is clear from the above that amount of reared brood fundamentally affects the conditions of the worker bees.

### 4.4. Hemolymph Storability

The results did not confirm that the determination of the THP content in hemolymphs is influenced by adding PTU or the storage temperature. We recommend using a temperature of −20 °C to prevent an incidental microbial effect. Time had a slight impact. However, time-dependent differences had similar variability to that which was inside the groups expressed by a coefficient of variation not exceeding 12%. We do not assume that proteases had any impact because the course of changes was increasing, possibly due to the increase in protein concentration caused by the evaporation of a diluent. Both older [8] and recent studies [17] determined bee THP content by analysing fresh hemolymph samples and used glutathione [8] or phenylthiourea [17] to prevent hemolymph melanization. If the melanization of the hemolymph sample during storage was to affect the protein assay, then the differences between the THP values in the stored samples and the fresh sample should be lower in the PTU group. However, we found the opposite result. Therefore, the impact of PTU was not confirmed. The melanization had a negligible impact on the spectrophotometric Bradford protein assay, probably due to the considerably high dilution of the stored hemolymph samples, and, therefore, the intensity of hemolymph darkening was mitigated. The situation in undiluted hemolymphs would certainly be different.

## 5. Conclusions

This study deepens current knowledge about the development of honeybee physiological markers during the year and the storability of hemolymph samples before their analysis. Significant seasonal impact on workers’ biomarkers (HG size and THP content) development was found. The highest biomarker values were monitored in the late summer workers of the same chronological age, and a gradual seasonal increase from spring to late summer was recorded in all chronological age categories. Moreover, the present study brings new insights into the spatial distribution of workers within the hive nest. The same-chronological-age workers were found in both upper and lower hive positions according to their biological age within the entire season. The biologically younger workers appeared in the upper parts, whereas the biologically older ones appeared in the lower parts of the hive nest. Moreover, a strong correlation in the THP content was observed between the same age workers in the upper and lower hive positions. The present results expand the existing findings in the field of worker distribution within the hive nest during their chronological aging. As the workers aged, they disappeared from the top parts of the hive nest and shifted to the lower parts of the hive. This finding contradicts the general guidelines for beekeepers to establish starters, package bees and mating hives/nuclei where young bees are needed, which generally recommend taking workers (nurses) from the brood chamber. Regarding this result, we propose preferably taking young bees from the honey chamber for these purposes.

It can be concluded that it is essential to perform physiological analyses of workers of known age sampled at different positions within the hive nest to achieve a precise result that reliably reflects the condition of the colony as a whole. Further research into physiological markers indicating the biological age of bees, and, thus, of the entire colony, is required.

## Figures and Tables

**Table 1:** Number of workers in the final samples.

Seasonal Cohort	Workers’ Age (Weeks) Number of Sampled Workers in Each Colony Strength: Weak + Medium + Strong, Respectively					
	0	1	2	3	4	5
Spring	5 + 5 + 5	5 + 10 + 10	5 + 10 + 10	5 + 10 + 10	5 + 5 + 5	0
Early summer	5 + 5 + 5	5 + 10 + 10	10 + 10 + 10	10 + 10 + 10	5 + 5 + 5	5 + 3 + 5
Late summer	0 + 7 + 7	0 + 14 + 14	0 + 14 + 14	0 + 14 + 14	0 + 7 + 4	0 + 7 + 0

**Table 2:** THP content (µg/µL) in fresh (0 h = control group) and stored hemolymph samples (**H**, for 24 h and 1–4 weeks), with phosphate buffer (H+PBS) or PBS with N-phenylthiourea (H+PBS+PTU) in relation to the storage time at all temperatures; *p*-value for the *t*-test—significant ones are in bold; c_v_-coefficient of variation.

Sample Composition	Parameter	0 h (n = 6)	24 h (n = 18)	1 Week (n = 18)	2 Weeks (n = 18)	4 Weeks (n = 18)
H+PBS	*p*-Value		0.808	0.851	0.887	**0.006**
	$\bar{x}$ ± SEM	22.8 ± 0.3	22.7 ± 0.3	22.6 ± 0.5	22.9 ± 0.3	25.3 ± 0.5
	c_v_	3.6%	6.1%	9.1%	5.5%	7.6%
H+PBS+PTU	*p*-Value		**0.015**	**0.020**	0.532	0.133
	$\bar{x}$ ± SEM	21.3 ± 0.5	23.3 ± 0.4	22.8 ± 0.3	20.8 ± 0.4	23.1 ± 0.6
	c_v_	6.0%	7.5%	5.8%	8.6%	11.4%

## Data Availability

Data are contained within the article.
